# Valorization of Sunflower Seed Husks into Cellulose Nanofibrils and Nanocrystals for Sustainable Hydrogel Adsorbents in Heavy Metal Removal

**DOI:** 10.3390/polym18151903

**Published:** 2026-08-03

**Authors:** Ainur K. Battalova, Kydyrmolla Akatan, Ansagan Demeukhan, Esbol Shaimardan, Nariman R. Kaiyrbekov, Zhandos R. Sagdollin, Ainur K. Kabdrakhmanova, Sana K. Kabdrakhmanova, Bhanumathyamma Deepa, Sabu Thomas

**Affiliations:** 1 National Scintific Laboratory Collective Use, S. Amanzholov East Kazakhstan University, 55 Kazakhstan Str., 070000 Ust-Kamenogorsk, Kazakhstan; kidirmolla@gmail.com (K.A.); demeuhanansagan@gmail.com (A.D.); narimankayrbekov@gmail.com (N.R.K.); zhandossagdollin@gmail.com (Z.R.S.); 2 Scientific Center of Composite Materials, 79 Nurmakov Str., 050000 Almaty, Kazakhstan; esbol_shay@mail.ru (E.S.); ainurkabdrahmanova@mail.ru (A.K.K.); 3 International R&D Center for Advanced Functional Materials and Their Composites, Satbayev University, 22 Satbayev Str., 050000 Almaty, Kazakhstan; 4 School of Energy Materials, Mahatma Gandhi University, Kottayam 686560, Kerala, India; deepabkaleeckal@gmail.com (B.D.); sabuthomas@mgu.ac.in (S.T.)

**Keywords:** hydrogel, cellulose, cellulose nanocrystalline, cellulose nanofibers, biodegradation, water retention, swelling properties, adsorption, wastewater treatment, metal ion removal

## Abstract

The application of cellulose-based nanocomposite sorbents as environmentally friendly materials for water purification has emerged as an important and rapidly developing research area. In this context, cellulose nanofibrils (CNFs) and cellulose nanocrystals (CNCs) were successfully extracted from microcrystalline cellulose (MCC) derived from sunflower seed husks (SFHs) and comprehensively characterized. The obtained nanocellulosic materials were subsequently utilized to fabricate nanocomposite hydrogels, designated as HG_CNF_ and HG_CNC_. The results revealed distinct structural and physicochemical characteristics of CNFs and CNCs, which significantly affected the morphology, swelling behavior, and stability of the resulting hydrogels. Swelling experiments conducted under various environmental conditions demonstrated that HG_CNF_ exhibited higher water uptake and swelling capacity than HG_CNC_. Both hydrogels showed maximum swelling under near-neutral conditions (pH ≈ 6.5) and exhibited pronounced sensitivity to changes in ionic strength and solvent polarity. Furthermore, adsorption studies confirmed the effective removal of Cu^2+^ ions by both hydrogels, with HG_CNC_ exhibiting a slightly higher adsorption capacity than HG_CNF_; the degree of sorption was 51.5%. These findings demonstrate that nanocellulose-based hydrogels possess tunable physicochemical properties and considerable potential as sustainable sorbent materials for water treatment, while also offering promising applications in environmental remediation, controlled drug delivery, and biomedical engineering.

## 1. Introduction

The growing global demand for sustainable bio-based materials has prompted considerable research into the valorization of agricultural waste streams as renewable sources for the production of high-value nanocellulose materials [1,2,3,4,5]. Among various lignocellulosic feedstocks, sunflower seed husks (SFHs) represent an abundant yet underutilized byproduct of the oilseed industry, offering significant potential for valorization [6,7,8,9]. Although previous studies have explored biomass sources such as soybean hulls and wheat husks for the development of functional hydrogel adsorbents [10,11,12], the specific potential of SFHs as a precursor for producing distinct nanocellulose morphologies, namely cellulose nanofibrils (CNFs) and cellulose nanocrystals, remains largely unexplored. To address this knowledge gap, a systematic comparative evaluation of cellulose nanofibrils (CNFs) and cellulose nanocrystals (CNCs) derived from sunflower seed husks (SFHs) was conducted, focusing on their structural, morphological, and functional properties. The obtained nanocellulosic materials were subsequently engineered into hydrogels and assessed for their potential in heavy metal remediation.

Previous studies [13,14,15] have demonstrated the feasibility of using cellulose-based hydrogel sorbents derived from agricultural waste for the efficient removal of heavy metal ions from contaminated water. These hydrogels exhibited remarkable adsorption performance, with maximum adsorption capacities reaching 2240 mg g^−1^ and removal efficiencies as high as 98.86%, highlighting their significant potential for sustainable water treatment applications.

Furthermore, earlier research studies mainly focused on homogenous extraction methods that produce a single type of nanocellulose and the development of film-based materials using these single nanocellulose forms derived from SFHs [16,17,18].

However, this study presents the design and synthesis of highly efficient, three-dimensional hydrogel networks based on CNFs and CNCs, highlighting the influence of nanocellulose type on the morphology, adsorption kinetics, crystallinity, thermal stability, mechanical stability and swelling behavior. By optimizing both the extraction process and hydrogel crosslinking mechanisms, this work demonstrates a scalable route for the conversion of agricultural residues into high-value, eco-friendly materials with significant potential for advanced wastewater treatment. The three-dimensional nanocellulose networks with enhanced surface functionality promote rapid ion exchange and strong chelation, essential for efficient heavy metal sequestration in complex aqueous environments [19,20,21,22,23].

In this study, cellulose nanofibrils (CNFs) and cellulose nanocrystals (CNCs) were produced from microcrystalline cellulose (MCC) derived from sunflower seed husks (SFHs) via formic acid and sulfuric acid hydrolysis, respectively. The proposed approach offers a cost-effective and sustainable route for the production of nanocellulosic materials from agricultural residues. The resulting CNFs and CNCs were subsequently employed as precursors for the fabrication of nanocellulose-based hydrogels. This work provides valuable insights into the influence of key physicochemical properties, including crystallinity, surface charge, and network architecture, on the adsorption behavior and reusability of SFH-derived CNF- and CNC-based hydrogels. Compared with conventional hydrogels prepared from wood-derived or other lignocellulosic nanocellulose sources, the developed materials demonstrate competitive performance while promoting the valorization of agricultural waste streams. Furthermore, this study contributes to bridging the gap between laboratory-scale synthesis and practical implementation by developing multifunctional adsorbents with high adsorption efficiency and reusability for environmental remediation and wastewater treatment applications.

A comprehensive suite of analytical techniques was employed to investigate the structural, physicochemical, and functional properties of the CNF- and CNC-based hydrogels. Fourier-transform infrared spectroscopy (FTIR) was used to elucidate chemical structures, X-ray diffraction (XRD) to determine crystallinity, thermogravimetric analysis (TGA) to evaluate thermal stability, and zeta potential measurements to assess surface charge and colloidal stability. Furthermore, the swelling behavior of the hydrogels under various environmental conditions and their Cu^2+^ adsorption performance were systematically examined to establish relationships between material characteristics and functional properties. This integrated characterization strategy provides valuable insights into the design, optimization, and development of sustainable nanocellulose-based hydrogels derived from sunflower seed husks (SFHs) for environmental remediation and wastewater treatment applications.

## 2. Materials and Methods

### 2.1. Materials

Sunflower husks (SFHs) of the Belosnezhka variety were supplied by the East Kazakhstan Agricultural Experimental Station LLP (Ust-Kamenogorsk, Kazakhstan). Hydrogen peroxide (15%, H_2_O_2_), acetic acid (≥44%, CH_3_COOH), potassium permanganate (≥99%, KMnO_4_), absolute ethanol (≥99.5%, C_2_H_5_OH), sulfuric acid (≥98%, H_2_SO_4_), formic acid (≥96%, HCOOH), acrylamide (≥99%, CH_2_=CHCONH_2_), N,N′-methylenebis(acrylamide) (≥99%), ammonium persulfate (≥98%, (NH_4_)_2_S_2_O_8_), disodium hydrogen phosphate (≥99%, Na_2_HPO_4_), sodium dihydrogen phosphate (≥99%, NaH_2_PO_4_), sodium chloride (≥99%, NaCl), acetone (≥99.5%, CH_3_COCH_3_), orthophosphoric acid (98%, H_3_PO_4_), sodium hydroxide (≥99%, NaOH), copper nitrate (≥99%, Cu(NO_3_)_2_), potassium dichromate (≥99%, K_2_Cr_2_O_7_), sodium thiosulfate (99%, Na_2_S_2_O_3_), potassium iodide (≥99%, KI), and starch were purchased from Sigma-Aldrich (Bangalore, India). Unless otherwise specified, all chemicals were of analytical grade and used as received without further purification.

### 2.2. Methods

#### 2.2.1. Preparation of Peroxyacetic Acid (PAA)

Peroxyacetic acid (PAA) was prepared according to the procedure previously reported in the literature [24,25,26,27,28,29]. The resulting PAA solution was stored at 5 ± 0.5 °C until further use.

#### 2.2.2. Preparation of Microcrystalline Cellulose (MCC) from Sunflower Seed Husks (SFHs) via Organosolv Oxidation

Microcrystalline cellulose (MCC) was isolated from sunflower seed husks (SFHs) following the organosolv oxidation procedure described in previous studies [24,25,26,27,28,29]. Briefly, 10 g of SFHs was mixed with PAA at a solid-to-liquid ratio of 1:12 (g/mL). Delignification was carried out in a round-bottom flask equipped with a reflux condenser at 90 ± 2 °C under continuous stirring for 40 min.

After the reaction, the obtained MCC was cooled to 25 ± 2 °C, separated by filtration, and thoroughly washed with distilled water until neutral pH was attained. The purified MCC was then dried at 80 ± 2 °C for 6 h to constant weight. The dried product was weighed using an analytical balance with a precision of ±0.0001 g, and the MCC yield was calculated according to Equation (1) [24]:

Yield = (m_SFH_ − m_MCC_)/m_SFH_ × 100% (1)

where m_SFH_ is the mass of SFH, g; m_MCC_ is the mass of the obtained MCC, g.

#### 2.2.3. Preparation of Cellulose Nanofibrils (CNFs) by Formic Acid Hydrolysis

Cellulose nanofibrils (CNFs) were prepared from microcrystalline cellulose (MCC) according to the method reported by Battalova et al. [18]. Briefly, 1 g of MCC was dispersed in 88% formic acid at an MCC-to-acid ratio of 1:20 (g/mL). The hydrolysis reaction was conducted in a round-bottom flask equipped with a reflux condenser at 90 ± 2 °C for 6 h under continuous stirring.

Upon completion of the reaction, the suspension was cooled to 25 ± 2 °C and neutralized by repeated washing with distilled water. The resulting dispersion was centrifuged using a ZT-TG16 centrifuge (Hunan Zetop Medical Co., Ltd., Changsha, China) at 16,000 rpm, followed by a second centrifugation step at 5000 rpm to facilitate the separation and purification of the nanofibrils. The purified CNF suspension was subsequently ultrasonicated using a Scientz 3000F ultrasonic homogenizer (Ningbo Scientz Biotechnology Co., Ltd., Ningbo, China) operating at 40 kHz for 15 min to improve fibril dispersion. The suspension was then freeze-dried in an LGJ-12S lyophilizer (Beijing Songyuan Huaxing Biotechnology Co., Ltd., Guangzhou, China) to obtain dry CNF powder.

The CNF yield was calculated according to Equation (2) [18]:

Yield = m_CNF_/m_MCC_ × 100% (2)

where m_MCC_ is the mass (g) of the MCC used for hydrolysis and m_CNF_ is the mass (g) of the obtained cellulose nanofibrils.

The dry mass of the CNFs was determined using an LV 210-A analytical balance (Sartogosm, Saint Petersburg, Russia) with a precision of ±0.0001 g.

#### 2.2.4. Preparation of Cellulose Nanocrystals (CNCs) by Sulfuric Acid Hydrolysis

Cellulose nanocrystals (CNCs) were prepared from the previously obtained microcrystalline cellulose (MCC) through sulfuric acid hydrolysis following the procedure described in the literature [28]. Briefly, 1 g of MCC was gradually added to a 60 wt% H_2_SO_4_ solution at an MCC-to-acid ratio of 1:10 (g/mL), while maintaining the reaction temperature between 0 and 5 °C. After thorough mixing to obtain a homogeneous gel-like suspension, 25 mL of deionized water was added. The mixture was subsequently hydrolyzed in a thermostatically controlled water bath at 40 ± 2 °C under mechanical stirring at 500 rpm for 1 h.

The isolation and purification of CNCs were carried out in two stages. In the first stage, phytomelanin impurities were removed by centrifugation using a Centrifuge 5427R (Eppendorf, Hamburg, Germany) at 1500 rpm for 5 min, resulting in the sedimentation of pigment-rich particles. This purification step was repeated twice to ensure complete removal of the pigment. In the second stage, the suspension was centrifuged at 8000 rpm for 15 min, and the resulting CNC suspension was subsequently purified by dialysis against deionized water using Visking dialysis tubing (molecular weight cut-off, MWCO: 12,000–14,000 Da, Medicell Membranes Ltd., London, UK) for seven days until a neutral pH was achieved.

The CNC yield was determined according to Equation (3) [27]:

Yield = m_CNC_/m_MCC_ × 100% (3)

where m_MCC_ is the mass (g) of the MCC subjected to hydrolysis and m_CNC_ is the mass (g) of the obtained cellulose nanocrystals.

#### 2.2.5. Synthesis of Nanocellulose-Based Hydrogels

Nanocellulose-based hydrogels were synthesized according to the procedure reported in the literature [26]. Briefly, 1% 5 mL of CNF or CNC suspension was transferred into a cylindrical plastic mold, followed by the addition of 0.125 g of acrylamide (AAm) as the functional monomer, 10 mg of N,N′-methylenebisacrylamide (MBA) as the crosslinking agent, and 10 mg of ammonium persulfate (APS) as the radical initiator. The reaction mixture was stirred continuously until a homogeneous precursor solution was obtained.

**Figure 1:**
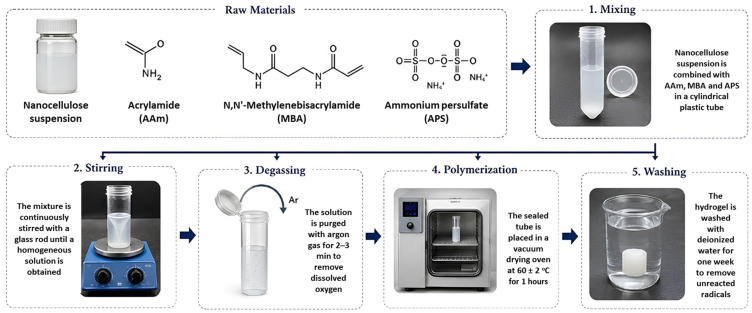
Hydrogel synthesis scheme.

To minimize oxygen inhibition of the free-radical polymerization process, the mixture was purged with argon for 2–3 min. Subsequently, polymerization was carried out in a DC-80 vacuum drying oven (Grodtorgmash, Minsk, Belarus) at 60 ± 2 °C for 1 h (Figure 1). Following gel formation, the hydrogels were immersed in deionized water for one week, with periodic water replacement, to remove residual monomers, initiator fragments, and other soluble impurities.

For clarity, the hydrogels prepared from cellulose nanofibrils and cellulose nanocrystals were designated as HG_CNF_ and HG_CNC_, respectively.

#### 2.2.6. Particle Size and Zeta Potential Analysis

The average particle size and zeta potential (ζ-potential) of the nanocellulosic materials were determined by dynamic light scattering (DLS) using a Zetasizer Nano ZS90 instrument (Malvern Panalytical, Mavern, UK). Prior to analysis, aqueous nanocellulose suspensions (2 wt%) were dispersed in an ultrasonic bath (U-sonic UZTA-0.15/22-0, Alena, Saint Petersburg, Russia) operating at a frequency of 30 kHz for 10 min to ensure homogeneous dispersion.

For each sample, measurements were performed in triplicate using three independently prepared suspensions. The results are reported as the mean value ± standard deviation.

#### 2.2.7. Scanning Electron Microscopy (SEM)

The surface morphology of the MCC, nanocellulose samples (CNF and CNC), and the corresponding hydrogels was examined using a Quanta 200i 3D scanning electron microscope (SEM) (FEI™, Eindhoven, The Netherlands). Micrographs were acquired in high-vacuum mode using a secondary electron detector at an accelerating voltage of 15 kV. Prior to imaging, the hydrogel samples were sputter-coated with a thin layer of gold to enhance surface conductivity and minimize charging effects during electron beam exposure. The specimens were then mounted on aluminum stubs using conductive carbon tape.

#### 2.2.8. Fourier-Transform Infrared (FTIR) Spectroscopy

Fourier-transform infrared (FTIR) spectra were recorded using an FT-801 spectrometer (Simex, Novosibirsk, Russia). Measurements were performed in the wavenumber range of 4000–400 cm^−1^ with a spectral resolution of 1 cm^−1^ at 25 °C. Spectra were collected using an attenuated total reflectance (ATR) accessory based on the internal reflection technique, and each spectrum was obtained by averaging 100 scans.

#### 2.2.9. X-Ray Diffraction (XRD) Analysis

The crystalline structure of the samples was investigated using an X’Pert PRO diffractometer (Malvern Panalytical, Almelo, The Netherlands) equipped with monochromatic Cu Kα radiation (λ = 0.1542 nm). Diffraction patterns were recorded in reflection mode over a 2θ range of 10–45° with a step size of 0.02°. The X-ray tube was operated at 45 kV and 30 mA, with a counting time of 0.5 s per step. Measurements were performed using an aluminum rectangular multi-purpose sample holder (PW1172/01). Phase identification and diffraction pattern analysis were conducted using the ICDD PDF-4/AXIOM database.

The crystallinity index (CI) was calculated using Equation (4) [30]:

CI = (Acryst/Acrys + Aamorph) × 100%(4)

where Acryst is the integrated area corresponding to the crystalline regions of cellulose and Aamorph is the integrated area associated with the amorphous fraction.

The crystallite size (CSL) was estimated using the Scherrer equation (Equation (5)):

CSL = kλ/βcos θ(5)

where k is the Scherrer shape factor (0.89) [31]; λ is the X-ray wavelength (0.1542 nm); β is the full width at half maximum (FWHM) of the (200) diffraction peak expressed in radians, and θ is the corresponding Bragg diffraction angle.

#### 2.2.10. Thermogravimetric Analysis (TGA)

The thermal stability and decomposition behavior of the samples were evaluated using a LabSys Evo thermogravimetric analyzer (Setaram, Lyon, France) under an argon atmosphere. Measurements were performed over a temperature range of 30–700 °C at a heating rate of 10 °C min^−1^. Approximately 25 ± 2 mg of each sample was used for the analysis.

#### 2.2.11. UV–Visible Spectroscopy

The optical absorption spectra of the aqueous samples were recorded using a PE-5400UV UV–visible spectrophotometer (Ecroskhim, Saint Petersburg, Russia). Measurements were conducted over the wavelength range of 190–1000 nm at a scanning rate of 240 nm min^−1^ and a spectral resolution of 1 nm. A quartz cuvette with an optical path length of 10 mm was used for all measurements.

#### 2.2.12. Inductively Coupled Plasma Mass Spectrometry (ICP–MS)

Elemental analysis was performed using an Agilent 7500cx inductively coupled plasma mass spectrometer (ICP–MS; Agilent Technologies, Santa Clara, CA, USA). Instrument calibration was conducted using certified multi-element standard solutions prepared by serial dilution of stock standards supplied by Inorganic Ventures (Christiansburg, VA, USA). Based on the calibration results, calibration curves for each determined element were constructed in the instrument software (ChemStation program Rev. B.03.05). According to the calibration curves, the analysis results were expressed in g/L for liquid samples using the software. The analysis was carried out in full quantitative mode. The mass spectrometer operated in analytical reaction mode.

#### 2.2.13. Swelling Properties of the HG_CNF_, HG_CNC_ Hydrogels

##### Swelling Kinetics in Water

The equilibrium swelling behavior of the hydrogels was evaluated using a gravimetric method. Hydrogel samples were initially dried in a DC-80 vacuum oven (Grodtorgmash, Minsk, Belarus) at 70 °C to constant weight and weighed using an LV 210-A analytical balance (Sartogosm, Saint Petersburg, Russia) with a precision of ±0.0001 g. The dried samples were subsequently immersed in deionized water at room temperature until swelling equilibrium was attained. The swollen hydrogels were removed, gently blotted to remove excess surface water, and weighed immediately.

The swelling degree was calculated according to Equation (6):

α (%) = (m − m_0_)/m_0_ × 100% (6)

where α is the swelling degree (%), m is the mass (g) of the swollen hydrogel at equilibrium, and m_0_ is the initial dry mass (g) of the hydrogel.

##### pH Responsiveness

The pH-responsive behavior of the HG_CNF_ and HG_CNC_ hydrogels was investigated using buffer solutions with pH values ranging from 3.5 to 9.0. Hydrogel samples were cut into disk-shaped specimens and immersed in the respective buffer solutions for 24 h. After equilibration, the samples were removed, surface moisture was carefully removed, and the mass was recorded.

The relative swelling ratio in the buffer solutions was determined using Equation (7):

α (%) = m_pH_/m_w.s._ × 100% (7)

where α is the mass (g) of the hydrogel swollen in the buffer solution and m_w.s._ is the mass (g) of the hydrogel swollen in deionized water under equilibrium conditions.

##### Effect of Ionic Strength

The influence of ionic strength on the swelling behavior of the hydrogels was examined using aqueous NaCl solutions with concentrations ranging from 0.001 to 1.0 N. Disk-shaped hydrogel samples were immersed in the salt solutions for 24 h, after which their masses were determined using the analytical balance described above.

The relative swelling ratio in saline media was calculated according to Equation (8):

α (%) = m_i.s._/m_w.s._ × 100% (8)

where m_i.s._ is the mass (g) of the hydrogel equilibrated in the NaCl solution and m_w.s_ is the mass (g) of the hydrogel swollen in deionized water.

##### Effect of Organic Solvent Composition

The stability and swelling behavior of the hydrogels in mixed solvent systems were evaluated using water/ethanol mixtures with volumetric ratios of 10:0, 8:2, 6:4, 5:5, 4:6, 2:8, and 0:10 (*v*/*v*). Hydrogel samples were immersed in the solvent mixtures for 24 h, and the resulting mass changes were recorded to assess solvent-induced swelling or shrinkage.

The swelling ratio in the mixed solvent systems was calculated using Equation (9):

α (%) = m_org_/m_w.s._ × 100% (9)

where m_org_ is the mass (g) of the hydrogel equilibrated in the water/ethanol mixture and m_w.s._ is the mass (g) of the hydrogel swollen in deionized water.

#### 2.2.14. Cu^2+^ Adsorption Experiments

The adsorption performance of HG_CNF_ and HG_CNC_ hydrogels toward Cu^2+^ ions was evaluated using a model aqueous copper nitrate (Cu(NO_3_)_2_) solution at room temperature (23 ± 2 °C). The initial concentration of the copper nitrate solution was 1.8757 g/L, corresponding to a Cu^2+^ ion concentration of 0.64 g/L. The solution pH was adjusted to 7.0 using 0.1 N KOH prior to the adsorption experiments.

Adsorption studies were initiated by contacting the hydrogel samples with the Cu^2+^ solution, and the uptake process was monitored from the first 5 min of exposure until adsorption equilibrium was attained. At predetermined time intervals, aliquots of the solution were collected for analysis to determine the residual Cu^2+^ concentration.

The concentration of Cu^2+^ ions before and after adsorption was quantified using UV-visible spectrophotometry and inductively coupled plasma mass spectrometry (ICP–MS). The adsorption capacity of the hydrogels was subsequently evaluated based on the decrease in Cu^2+^ concentration in the aqueous phase.

## 3. Results and Discussion

### 3.1. Characteristics of MCC, CNF and CNC Obtained from SFHs

Table 1 presents the physicochemical characteristics of MCC, CNFs, and CNCs derived from sunflower seed husks (SFHs), including yield, crystallinity index (CI), zeta potential, and average particle size. The isolated MCC exhibited a yield of 50.69 ± 2% and a CI of 68 ± 4%, reflecting the preservation of crystalline cellulose domains following the removal of amorphous constituents such as lignin and hemicellulose during organosolv treatment [32]. Its large particle size (length: 2372 ± 150 nm; width: 209 ± 30 nm) aligns with typical microscale cellulose dimensions.

The CNF sample produced at an MCC-to-formic acid ratio of 1:20 (g/mL) (CNF_1/20_) achieved a yield of 70.04 ± 2%. Compared with MCC, the crystallinity index increased from 68 ± 4% to 74.9 ± 4%, suggesting selective hydrolysis of amorphous cellulose fractions and consequent enrichment of the crystalline regions. This behavior is consistent with previous reports describing the preferential removal of less ordered cellulose domains during formic acid treatment [33]. The zeta potential of −15.8 mV suggests moderate colloidal stability, suggesting partial electrostatic stabilization of the suspension [34]. The average particle size of CNF was markedly reduced in width (35 ± 2 nm) but displayed increased length (4540 ± 50 nm), characteristic of the high-aspect-ratio fibrous morphology of nanofibrils.

In contrast, CNCs obtained by MCC/sulfuric acid hydrolysis of 1:10 g/mL (CNC_1/10_) showed a lower yield (35.6 ± 0.5%) due to strong acid conditions causing more extensive degradation. The CI was slightly lower (85.0 ± 4%) compared to CNF, which may be attributed to sulfate group introduction or partial crystalline disruption during acid hydrolysis [35,36,37]. The CNC suspension exhibited a more negative zeta potential (−23.6 mV), indicating enhanced colloidal stability resulting from the increased surface charge associated with sulfate ester groups introduced during sulfuric acid hydrolysis [38]. In addition, CNC particles displayed a substantially smaller average length (424.0 ± 150 nm) and a larger average width (129.2 ± 30 nm) than CNFs. These dimensional characteristics are consistent with the morphology of cellulose nanocrystals, which are composed of short, highly ordered crystalline domains with rod-like geometry.

**Figure 2:**
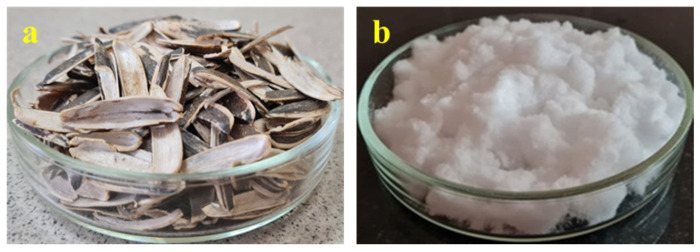
(**a**) SFH; (**b**) MCC obtained from SFH.

**Figure 3:**
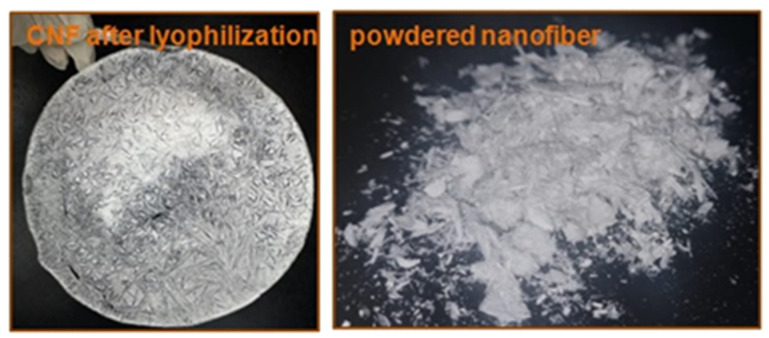
CNF obtained from MCC.

**Figure 4:**
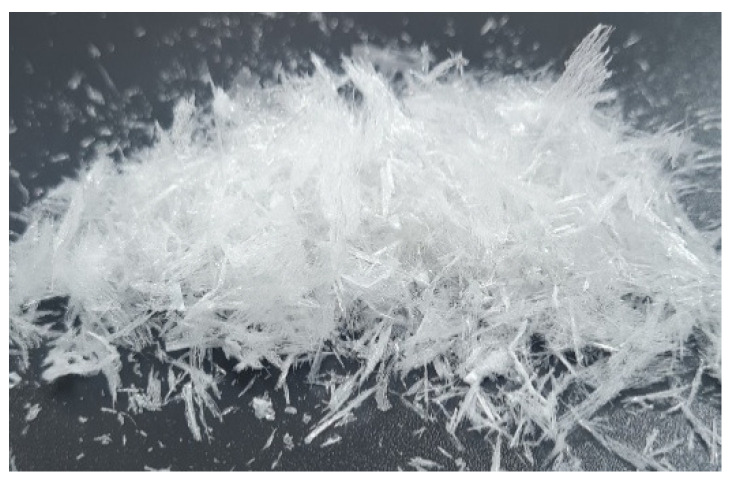
CNC obtained from MCC.

Figure 2, Figure 3 and Figure 4 visually confirm the morphological distinctions among MCC, CNF, and CNC. MCC particles appear as larger, irregular micro-sized cellulose fragments, while CNF images reveal elongated fibrillar networks. CNC images demonstrate discrete, rod-shaped nanoparticles consistent with their nanoscale dimensions.

**Figure 5:**
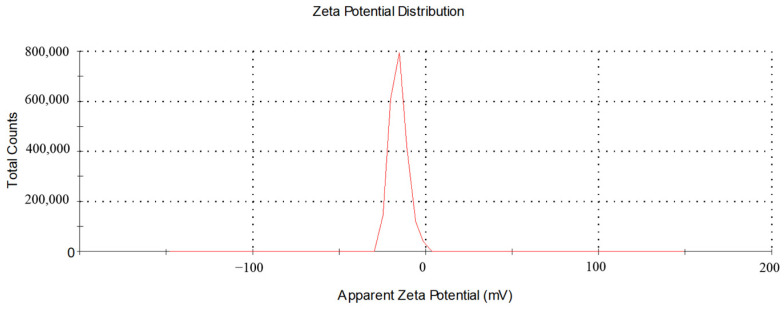
Zeta potential of CNFs produced by formic acid hydrolysis of MCC at an MCC-to-formic acid ratio of 1:20 (g/mL).

**Figure 6:**
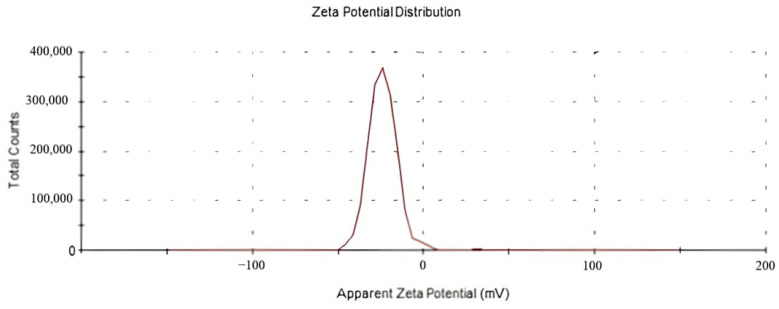
Zeta potential of CNCs produced by sulfuric acid hydrolysis of MCC at an MCC-to-H_2_SO_4_ ratio of 1:10 (g/mL).

**Figure 7:**
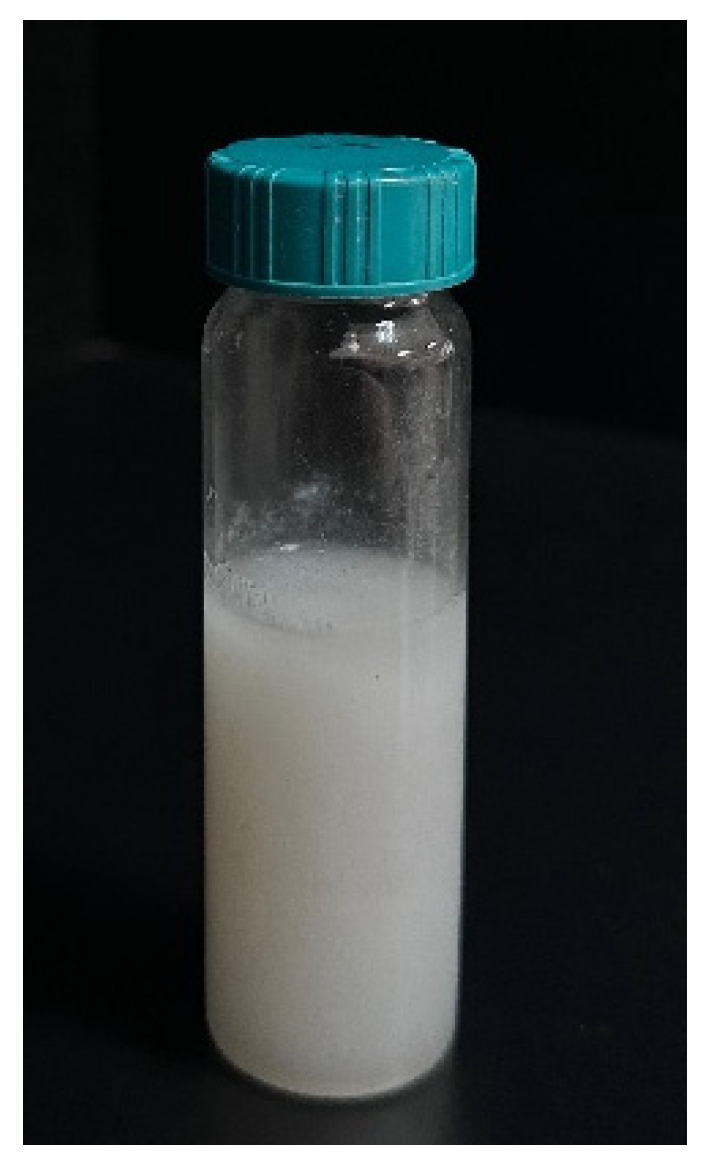
CNF suspension.

**Figure 8:**
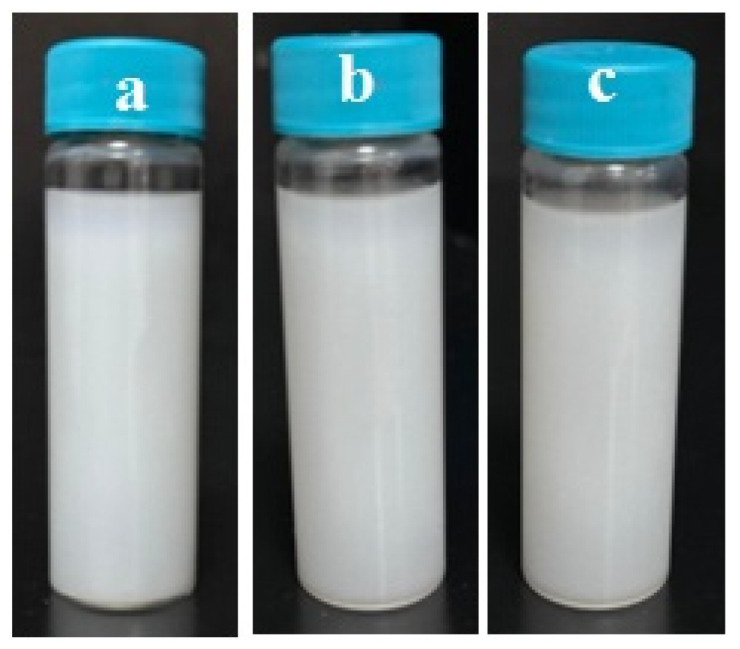
Visual appearance and sedimentation stability of the CNC suspension after storage for (**a**) 10, (**b**) 20, and (**c**) 30 days.

Figure 5 and Figure 6 quantify the average particle sizes of CNFs and CNCs, respectively, supporting the trend of CNFs having longer fibrils and CNCs exhibiting shorter, more uniform nanocrystals. Figure 7 and Figure 8 illustrate the stability of CNF and CNC suspensions over time, with CNC suspensions maintaining better dispersion stability up to 30 days, likely due to higher surface charge.

### 3.2. SEM Analysis of CNFs, CNCs, HG_cnf_, and HG_cnc_

**Figure 9:**
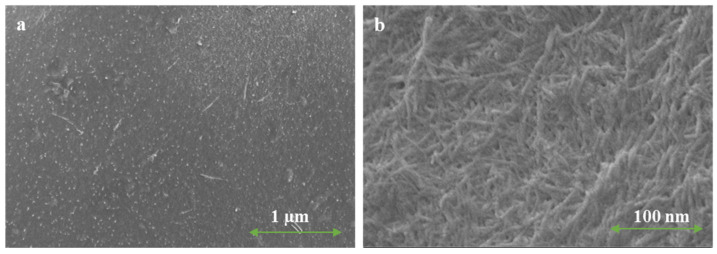
SEM image of CNFs: (**a**) SEM image at low magnification; (**b**) SEM image at high magnification showing the nanofibrillar structure.

**Figure 10:**
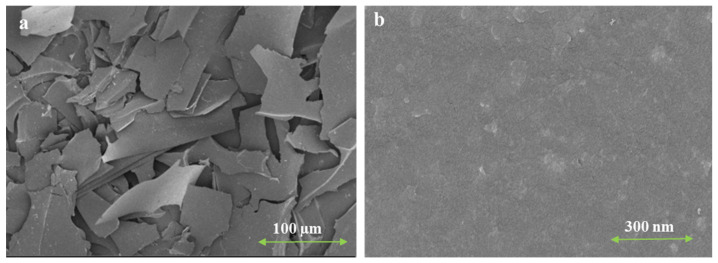
SEM image of CNCs: (**a**) SEM image of CNCs at low magnification (scale bar: 100 μm); (**b**) SEM image of CNCs at high magnification (scale bar: 300 nm).

SEM images (Figure 9 and Figure 10) provide detailed surface morphology of CNFs and CNCs. CNFs display entangled, web-like structures with high aspect ratios, corroborating their fibrous nature and potential for forming strong, flexible networks in composite applications [39]. CNCs exhibit more uniform, rod-like particles with smooth surfaces, indicating well-defined crystalline domains suitable for reinforcing materials where rigidity and mechanical strength are desired [40].

**Figure 11:**
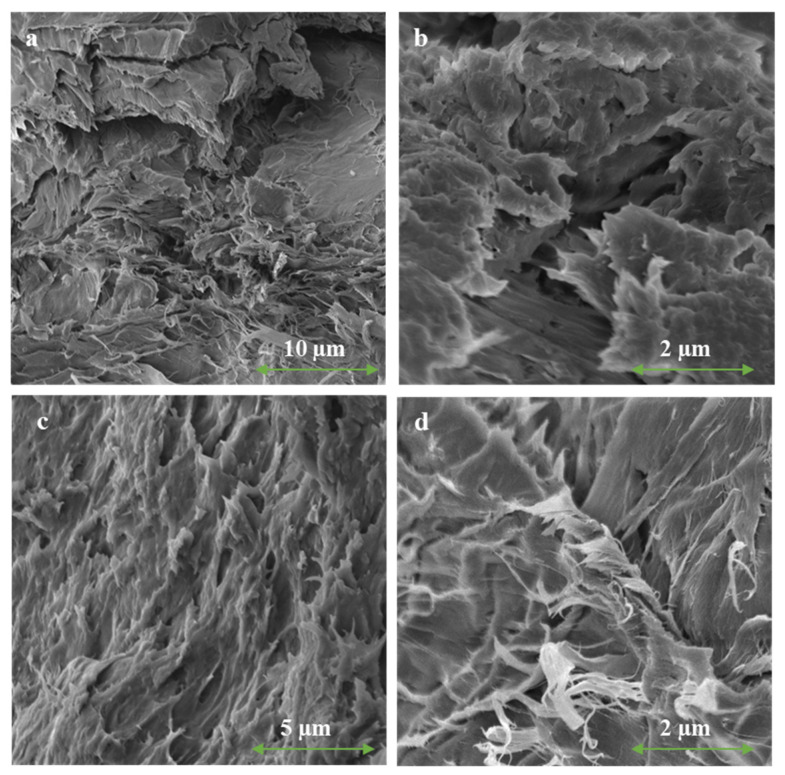
SEM images of hydrogels made of CNFs and CNCs. (**a**,**b**) HG_CNF_ and (**c**,**d**) HG_CNC_.

As shown in Figure 11, the SEM images revealed pronounced structural differences between the CNF- and CNC-based hydrogels. The HG_CNF_ hydrogel displayed a well-developed porous architecture consisting of interconnected fibrillar networks. Such a structure promotes efficient water retention and swelling behavior while contributing to mechanical resilience through nanofibril entanglement and extensive hydrogen-bond interactions within the polymer matrix. In contrast, CNC hydrogels (HG_CNC_) showed relatively denser and more compact structures, attributable to the higher crystallinity and intrinsic rigidity of CNC particles [41]. These morphological differences suggest that CNF-based hydrogels may be more suitable for applications requiring elasticity and swelling capacity, whereas CNC hydrogels are preferable for enhanced mechanical strength and stability.

Overall, the results revealed that acid hydrolysis parameters critically dictate the yield, crystallinity, particle size, surface charge, and morphology of cellulose derivatives from SFHs. The complementary properties of CNFs and CNCs such as high aspect ratio and flexibility versus rigidity and colloidal stability offer versatile opportunities for tailored applications in nanocomposites, hydrogels, and other advanced functional materials.

### 3.3. Fourier-Transform Infrared (FTIR) Spectroscopy

**Figure 12:**
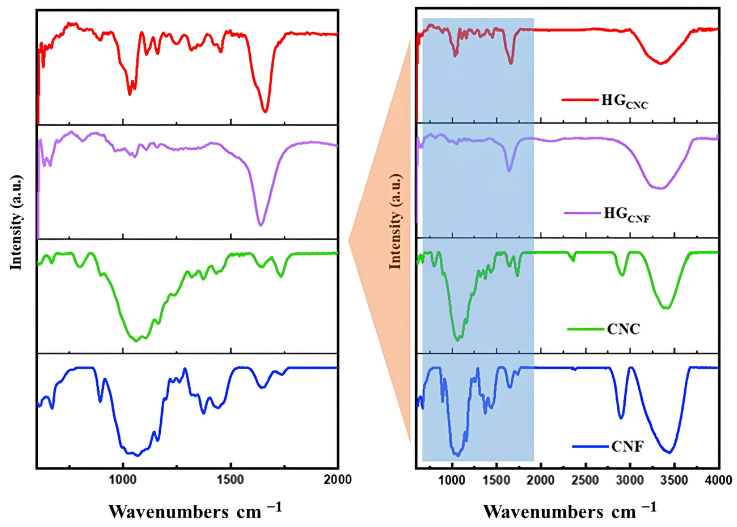
Comparative FTIR spectra of CNFs; CNCs; HG_CNF_; HG_CNC_.

The FTIR spectra of cellulose nanofibrils (CNFs), cellulose nanocrystals (CNCs), and their corresponding hydrogels (HG_CNF_ and HG_CNC_) provide valuable information regarding their chemical structures and the molecular interactions associated with hydrogel formation (Figure 12). The spectrum of CNFs exhibits the characteristic absorption bands of cellulose, including a broad band in the 3300–3500 cm^−1^ region attributed to O–H stretching vibrations, reflecting the extensive hydrogen-bonding network within the cellulose matrix [42]. The absorption band observed near 2900 cm^−1^ corresponds to C–H stretching vibrations, while the bands in the 1000–1200 cm^−1^ region are assigned to C–O–C and C–O stretching vibrations, confirming the preservation of the cellulose backbone structure following hydrolysis [43,44].

The FTIR spectrum of CNCs exhibits features similar to those of CNFs; however, the absorption bands are generally sharper and more intense, reflecting the higher degree of crystallinity resulting from the preferential removal of amorphous cellulose domains during sulfuric acid hydrolysis. Furthermore, the appearance of an absorption band near 1200 cm^−1^ is attributed to the presence of sulfate ester groups introduced during the hydrolysis process. These surface functional groups modify the surface chemistry of CNCs and may influence intermolecular and interfacial interactions in subsequent hydrogel networks [45].

In the FTIR spectrum of the synthesized hydrogel, the amide I bands of the polyacrylamide molecule were observed in the 1650–1670 cm^−1^ region, the amide II bands in the 1540–1600 cm^−1^ region, and the vibration band characteristic of the C–N bond was recorded at 1300–1450 cm^−1^ [46]. In addition, changes in the intensity of the broad band in the 3200–3500 cm^−1^ region, characteristic of the OH groups in CNC and CNF molecules, provide evidence for the formation of hydrogen bonds between the amide groups of polyacrylamide and the hydroxyl groups of CNC and CNFs [47]. The preservation of cellulose-specific C–O bands in the 1000–1160 cm^−1^ absorption region indicates that the nanocellulose framework did not lose its structural integrity during synthesis. Similar results have also been reported in previous studies [48,49].

### 3.4. XRD Analysis

**Figure 13:**
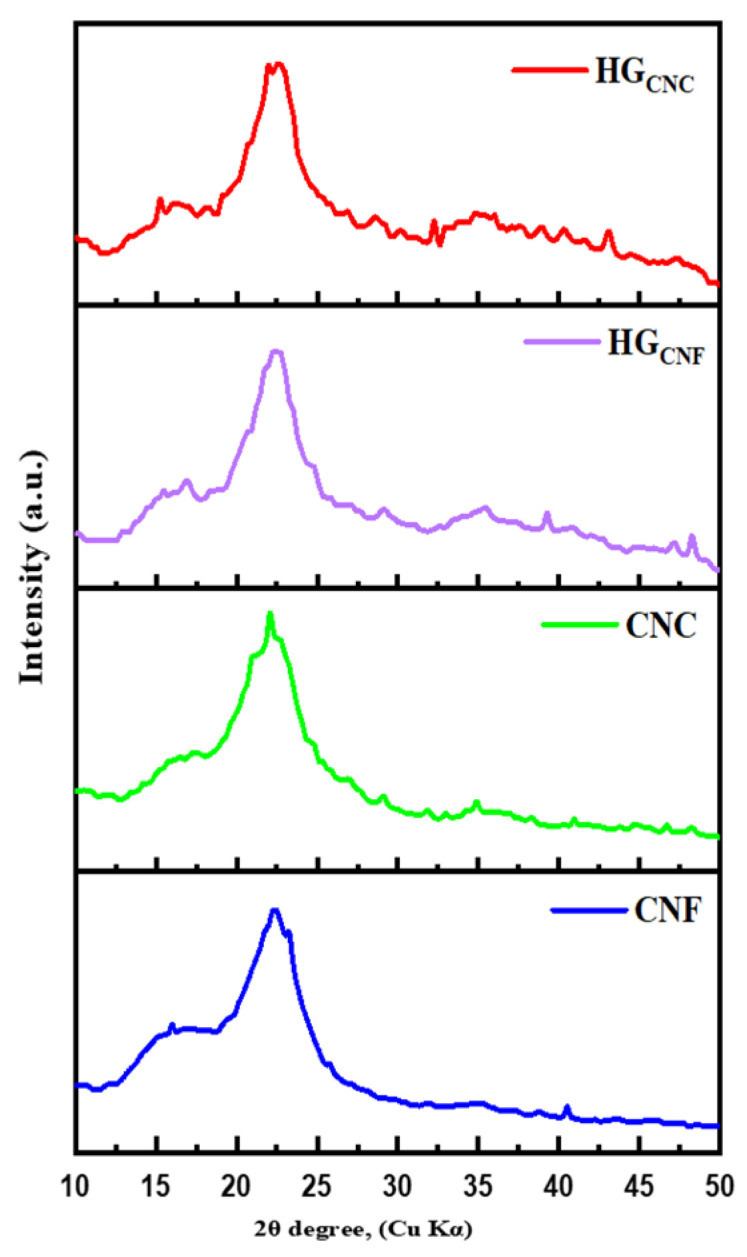
XRD of CNFs; CNCs; HG_CNF_; HG_CNC_.

The X-ray diffraction (XRD) patterns of the investigated samples are presented in Figure 13 and provide valuable information regarding their crystalline structure and structural organization. The CNF sample exhibited the characteristic diffraction peaks of cellulose I at approximately 2θ = 15.0°, 16.5°, and 22.5°, corresponding to the (1–10), (110), and (200) crystallographic planes, respectively. These diffraction features are indicative of the native cellulose I polymorph and reflect a semi-crystalline structure comprising both crystalline and amorphous domains [36]. CNC exhibits sharper and more intense peaks at the same positions, reflecting increased crystallinity due to selective removal of amorphous domains during acid hydrolysis [50]. In addition, the crystallinity index (CI) of the HG_CNF_ and HG_CNC_ samples was determined. The CI values were 58% for HG_CNF_ and 69% for HG_CNC_. Compared with the CI values of the initial CNF and CNC samples presented in Table 1, these values were found to be 1.3-fold and 1.2-fold lower, respectively. This can be attributed to the amount of CNFs and CNCs incorporated into the hydrogel matrix.

Hydrogels synthesized from CNFs and CNCs (HG_CNF_ and HG_CNC_) show broader and less intense diffraction peaks, indicating partial disruption of crystalline order. This decrease in crystallinity results from hydrogel network formation, where crosslinking and polymer entanglement introduce amorphous regions and hinder regular cellulose chain packing [51]. The crystallinity reduction is more pronounced in HG_CNC_, likely influenced by the initially higher crystallinity of CNCs and the nature of crosslinking interactions.

The FTIR and XRD data together indicate that hydrogel formation involves both chemical modification and physical restructuring. The introduction of crosslinkers or additives alters hydrogen bonding and introduces new functional groups, as evidenced by FTIR shifts and new peaks. Concurrently, the crystalline cellulose domains become partially disrupted or less ordered, as shown by XRD peak broadening and intensity reduction.

These changes impact the hydrogels mechanical properties, swelling behavior, and sorption capacities. Reduced crystallinity generally enhances flexibility and water uptake, while chemical modifications can tailor interactions with target molecules (e.g., metal ions in sorption applications). The presence of sulfate groups in CNCs and their retention or alteration during hydrogel synthesis may further influence surface charge and binding affinity.

Overall, the combined spectral analyses confirm successful hydrogel synthesis from CNFs and CNCs with distinct structural and chemical characteristics derived from their respective precursors and crosslinking processes.

### 3.5. Thermogravimetric Analysis (TGA)

**Figure 14:**
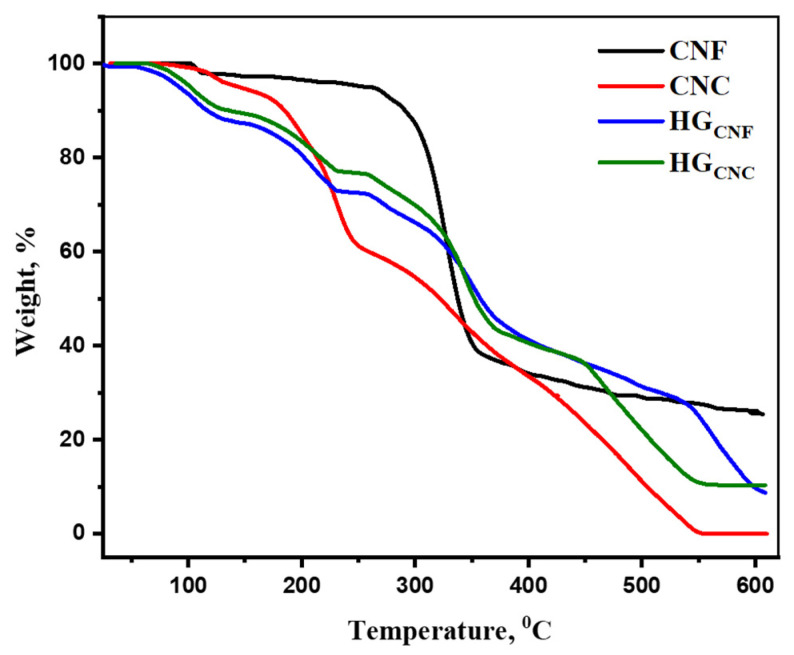
TGA curves of CNFs; CNCs; HG_CNF_; HG_CNC_.

The Thermogravimetric Analysis (TGA) curves (Figure 14) for CNFs, CNCs, HG_CNF_, and HG_CNC_ reveal clear differences in thermal stability among these materials.

An initial mass loss was observed for all samples in the temperature range of 30–150 °C. It amounted to 4% for CNF, 8% for CNC, 12% for HG_CNF_, and 11% for HG_CNC_. This is associated with the evaporation of physically adsorbed and bound water in the samples. The greater initial mass loss of the hydrogels can be explained by the retention of a larger amount of water in their three-dimensional network structure. Hydrogen bonding between the amide groups of polyacrylamide and the hydroxyl groups of nanocellulose contributes to the retention of water within the material [52].

In the next stage, depolymerization of all samples occurred. Thermal degradation of CNFs took place in the 290–400 °C range, with an approximate mass loss of 70%. In the case of CNCs, thermal degradation began at 196 °C and continued up to 400 °C, resulting in a mass loss of 60%. It was found that the thermal degradation of CNCs began earlier than that of CNF. This is attributed to the catalytic effect of sulfate ester groups formed during sulfuric acid hydrolysis, which reduce the thermal stability of cellulose [28,53].

Further decomposition of organic residues, such as polyacrylamide remnants and carbonized cellulose, was observed in the 400–600 °C range.

### 3.6. Swelling Kinetics of the CNF and CNC-Based Hydrogels

#### 3.6.1. Swelling Behavior of MCC-Based Hydrogels in Water

The swelling kinetics of HG_CNF_ and HG_CNC_ were systematically evaluated under various environmental conditions, revealing distinct behaviors relevant to their structural and functional properties.

**Figure 15:**
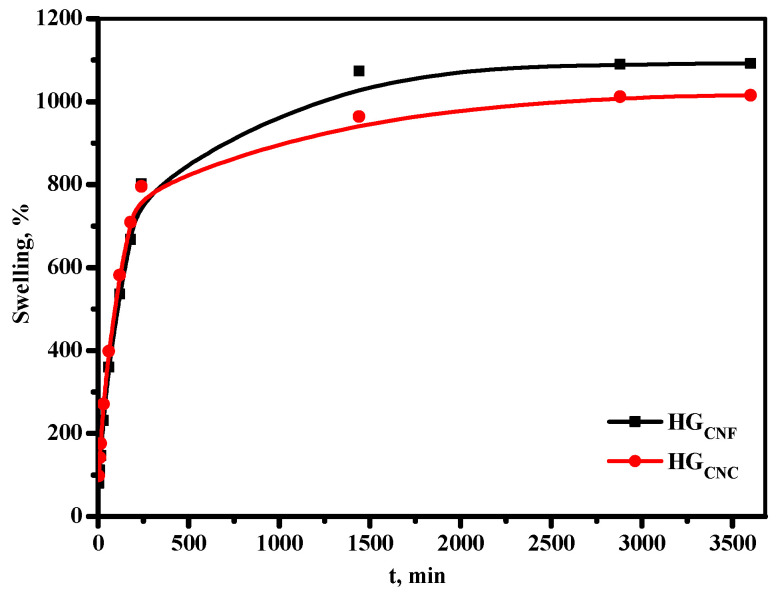
Water swelling kinetics of HG_CNF_ and HG_CNC_.

Figure 15 illustrates the swelling kinetics of HG_CNF_ and HG_CNC_ hydrogels in aqueous media. Both materials exhibited a rapid increase in swelling ratio during the first 60 min, after which equilibrium swelling was gradually established. HG_CNF_ displayed a higher equilibrium swelling ratio (12 g/g) than HG_CNC_ (10 g/g), indicating a greater water absorption capacity. This behavior is likely associated with the more open and interconnected network architecture of HG_CNF_, which promotes water penetration and enhances solvent retention.

#### 3.6.2. Swelling Kinetics of HG_CNF_ and HG_CNC_ Hydrogels Under Different pH Conditions

**Figure 16:**
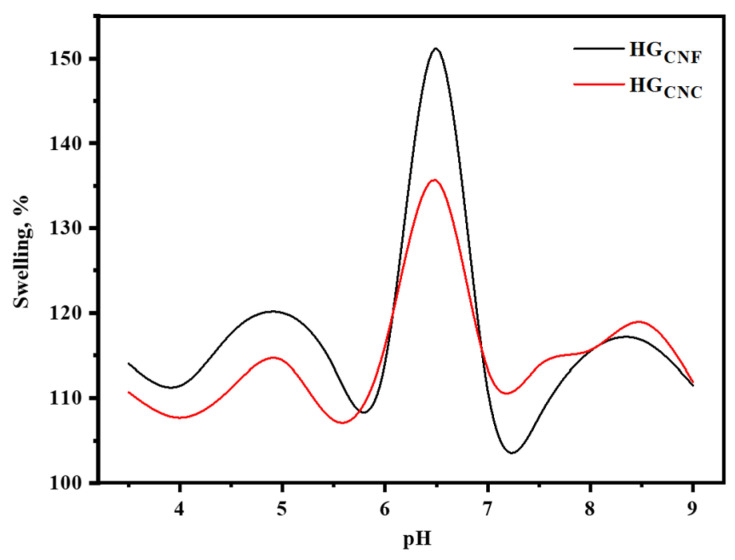
pH-responsive swelling behavior of HG_CNF_ and HG_CNC_ hydrogels.

Figure 16 shows that the swelling behavior of both hydrogels was strongly dependent on the pH of the surrounding medium. The highest swelling degree was observed at near-neutral pH (≈6.5). Under these conditions, the swelling degree of the HG_CNF_ and HG_CNC_ hydrogels was approximately 150% and 135%, respectively.

A pronounced decrease in swelling was observed in both acidic and alkaline media. At pH ≈ 3, the swelling degree of HG_CNF_ and HG_CNC_ was approximately 120% and 114%, respectively. Similar values were also recorded at pH ≈ 11. Such pH-dependent swelling behavior of the hydrogels is associated with changes in the ionization degree of functional groups within the network structure. Ionization of these functional groups affects the extent of hydrogel network expansion and its affinity for water molecules, thereby regulating the water absorption capacity of the material [54]. Similar results were reported in previous studies [55,56].

#### 3.6.3. Effect of Ionic Strength on Swelling Behavior of HG_CNF_ and HG_CNC_ Hydrogels

**Figure 17:**
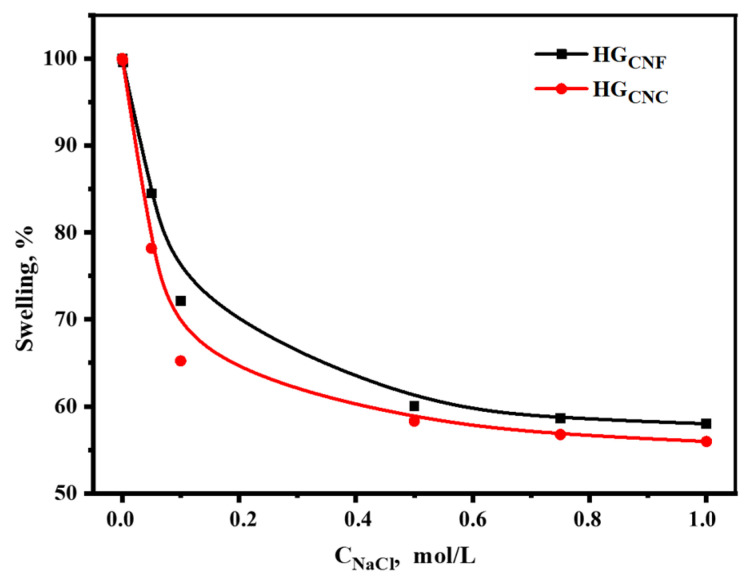
Variation in the swelling ratio of HG_CNF_ and HG_CNC_ hydrogels as a function of NaCl concentration.

To assess the effect of ionic strength, the swelling behavior of HG_CNF_ and HG_CNC_ hydrogels was examined in NaCl solutions with concentrations ranging from 0 to 1 M (Figure 17). At 0 M NaCl, swelling ratios align with those observed in water (~12 g/g for HG_CNF_ and ~10 g/g for HG_CNC_). Increasing salt concentration leads to a pronounced decrease in swelling, with values dropping to approximately 4 g/g for HG_CNF_ and 3.5 g/g for HG_CNC_ at 1 M NaCl. This reduction is attributed to ionic screening effects, which diminish osmotic pressure and restrict polymer network expansion [57].

#### 3.6.4. Effect of Organic Solvent Composition on Swelling Behavior of HG_CNF_ and HG_CNC_ Hydrogels

**Figure 18:**
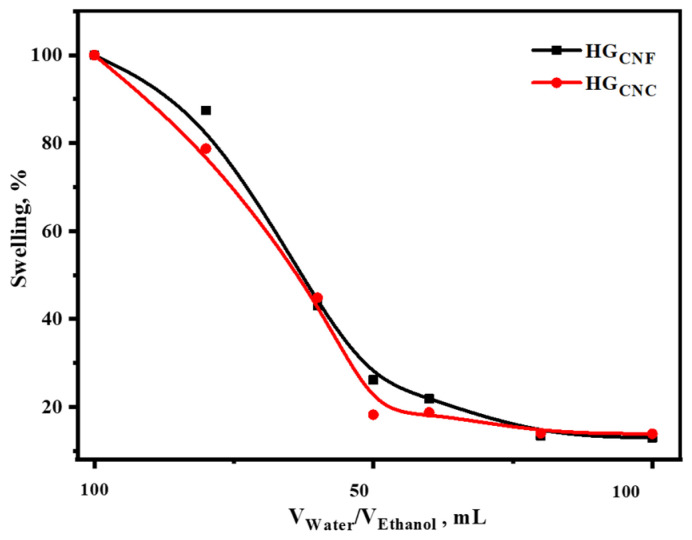
Swelling kinetics of HG_CNF_ and HG_CNC_ in ethanol solutions.

As shown in Figure 18, the swelling kinetics of HG_CNF_ and HG_CNC_ in ethanol solutions were significantly suppressed compared with those in water. Both hydrogels rapidly approached equilibrium, attaining relatively low swelling ratios of approximately 2.0 g/g for HG_CNF_ and 1.5 g/g for HG_CNC_ after 120 min. The decreased swelling capacity is likely associated with the reduced polarity of ethanol, which weakens polymer–solvent interactions and limits the penetration of solvent molecules into the hydrogel matrix.

In conclusion, HG_CNF_ consistently demonstrates superior swelling capacity relative to HG_CNC_ across all tested conditions, indicating its enhanced water retention and network flexibility. Both hydrogels are sensitive to environmental factors such as pH, ionic strength, and solvent polarity, which modulate their swelling behavior. These findings highlight the potential for tuning hydrogel properties for targeted applications such as controlled release, biomedical devices, or environmental remediation by manipulating external conditions.

### 3.7. Sorption Capacity of Hg_cnc_ and Hg_cnf_ for Cu^2+^

**Figure 19:**
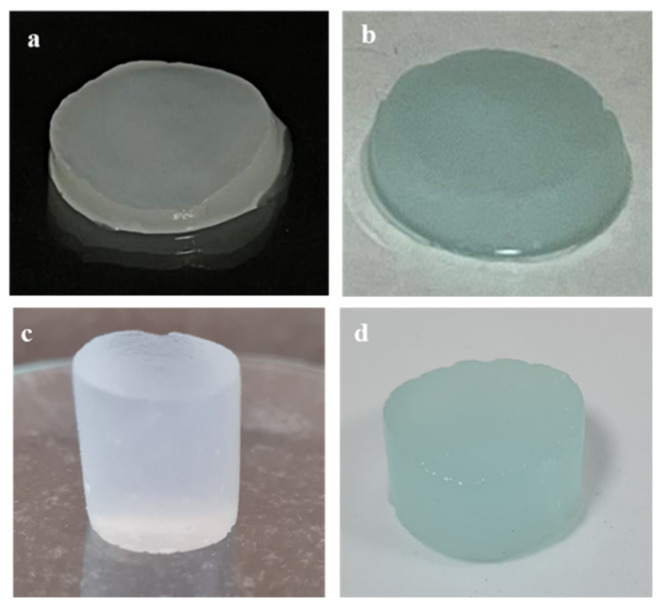
(**a**) Initial HG_CNC_; (**b**) HG_CNC_ after Cu^2+^ sorption; (**c**) initial HG_CNF_; (**d**) HG_CNF_ after Cu^2+^ sorption.

Regarding sorption capacity for Cu^2+^ ions (Figure 19 and Table 2), both hydrogels demonstrate effective adsorption. HG_CNC_ shows a Cu^2+^ concentration reduction from an initial value of 0.64 g/L to 0.31 g/L by UV spectroscopy and to 0.33 g/L by ICP-MS, corresponding to sorption degrees of 48.4% and 51.5%, respectively. HG_CNF_ reduces Cu^2+^ concentration to 0.30 g/L (UV) and 0.31 g/L (ICP-MS), with sorption degrees of 46.8% and 48.4%. The close agreement between UV and ICP-MS confirms measurement reliability.

The slightly higher sorption capacity of HG_CNC_ compared to HG_CNF_ may be due to its structural or chemical differences, such as increased surface area or a higher density of functional groups, which facilitate more effective Cu^2+^ ion binding.

The slightly higher Cu^2+^ sorption capacity of the HG_CNC_ hydrogel compared with HG_CNF_ can be explained by its structural and chemical features. Specifically, surface modification of CNCs with sulfate groups during sulfuric acid hydrolysis leads to an increase in the number of negative charges [28]. The comparative zeta potential values of MCC, CNCs, and CNFs are clearly presented in Table 1. This, in turn, promotes more effective binding of Cu^2+^ ions to the hydrogel surface and enhances their adsorption.

### 3.8. The Techno-Economic Feasibility

The synthesized hydrogels were shown to be producible at relatively low material cost. This is primarily due to the significant reduction in cost achieved by using cellulose nanofibrils (CNFs) and cellulose nanocrystals (CNCs) obtained from agricultural waste (SFHs) instead of commercial nanocellulose. Based on calculations using the costs of acrylamide, N,N′-methylenebisacrylamide, ammonium persulfate, and laboratory-produced CNFs/CNCs, the estimated production cost of the hydrogels was approximately USD 30 per kg.

The efficient utilization of renewable biomass waste and the possibility of producing hydrogels at low cost demonstrate the economic feasibility of the developed materials and their compliance with the principles of sustainable development. This indicates their potential for large-scale application in environmental protection, particularly in wastewater treatment technologies.

## 4. Conclusions

The conversion of MCC from SFHs into CNF and CNC via controlled acid hydrolysis yielded nanocellulose materials with distinct morphological and physicochemical characteristics. CNF exhibited higher crystallinity, a larger aspect ratio, and moderate colloidal stability, while CNC showed enhanced crystallinity and surface charge but smaller particle dimensions. Hydrogels fabricated from these nanocelluloses demonstrated markedly different swelling behaviors: HG_CNF_ possessed a more porous and flexible network facilitating higher swelling ratios and water retention, whereas HG_CNC_ formed denser structures with comparatively lower swelling. Both hydrogels were responsive to environmental factors, including pH, ionic strength, and solvent type, which modulated their swelling kinetics and equilibrium states. Thermal analysis confirmed improved stability upon hydrogel formation. Sorption studies validated the capacity of both hydrogels to effectively adsorb Cu^2+^ ions, with HG_CNC_ marginally outperforming HG_CNF_. The degree of sorption of the HG_CNC_ hydrogel was 51.5%, likely due to structural and surface chemistry differences. Overall, this study highlights the versatility of nanocellulose-based hydrogels for tailored functional applications, indicating the importance of precursor selection and environmental conditions in optimizing performance.

## Figures and Tables

**Table 1:** Crystallinity index (CI), crystallite size (CSL), and average particle size of CNF and CNC produced from MCC derived from SFHs via acid hydrolysis.

Specimens	Yield, %	CSL, nm	Crystallinity Index (CI), (%)	Zeta Potential, mV	Average Particle Size, nm	
					Length	Width
MCC	50.69 ± 2	2.9 ± 0.1	68 ± 4	-	2372 ± 150	209 ± 30
CNF_1/20_	70.04 ± 2	2.7 ± 0.1	74.9 ± 4	−15.8	4540 ± 50	35 ± 2
CNC_1/10_	35.6 ± 0.5	2.6 ± 0.1	85.0 ± 4	−23.6	424.0 ± 50	129.2 ± 30

**Table 2:** The sorption capacity of HG_CNC_ and HG_CNF_ hydrogels for Cu^2+^.

Samples	UV Spectroscopy		ICP-MS	
	Cu^2+^			
	Concentration, g/L	Sorption Degree, %	Concentration, g/L	Sorption Degree, %
HG_CNC_	0.31	48.4%	0.33	51.5%
HG_CNF_	0.30	46.8%	0.31	48.4%

## Data Availability

All data generated or analyzed during this study are included in this published article. Further inquiries can be directed to the corresponding authors.
